# Tidal Flushing Restores the Physiological Condition of Fish Residing in Degraded Salt Marshes

**DOI:** 10.1371/journal.pone.0046161

**Published:** 2012-09-27

**Authors:** Kimberly L. Dibble, Laura A. Meyerson

**Affiliations:** 1 Department of Natural Resources Science, University of Rhode Island, Kingston, Rhode Island, United States of America; 2 Department of Invasion Ecology, Institute of Botany, Academy of Sciences of the Czech Republic, Průhonice, Czech Republic; University of Hamburg, Germany

## Abstract

Roads, bridges, and dikes constructed across salt marshes can restrict tidal flow, degrade habitat quality for nekton, and facilitate invasion by non-native plants including *Phragmites australis*. Introduced *P. australis* contributes to marsh accretion and eliminates marsh surface pools thereby adversely affecting fish by reducing access to intertidal habitats essential for feeding, reproduction, and refuge. Our study assessed the condition of resident fish populations (*Fundulus heteroclitus*) at four tidally restricted and four tidally restored marshes in New England invaded by *P. australis* relative to adjacent reference salt marshes. We used physiological and morphological indicators of fish condition, including proximate body composition (% lipid, % lean dry, % water), recent daily growth rate, age class distributions, parasite prevalence, female gravidity status, length-weight regressions, and a common morphological indicator (Fulton’s K) to assess impacts to fish health. We detected a significant increase in the quantity of parasites infecting fish in tidally restricted marshes but not in those where tidal flow was restored to reduce *P. australis* cover. Using fish length as a covariate, we found that unparasitized, non-gravid *F. heteroclitus* in tidally restricted marshes had significantly reduced lipid reserves and increased lean dry (structural) mass relative to fish residing in reference marshes. Fish in tidally restored marshes were equivalent across all metrics relative to those in reference marshes indicating that habitat quality was restored via increased tidal flushing. Reference marshes adjacent to tidally restored sites contained the highest abundance of young fish (ages 0–1) while tidally restricted marshes contained the lowest. Results indicate that *F. heteroclitus* residing in physically and hydrologically altered marshes are at a disadvantage relative to fish in reference marshes but the effects can be reversed through ecological restoration.

## Introduction

It is well established that fish and swimming crustaceans (termed “nekton”) use vegetated intertidal salt marsh habitats for refuge, feeding, as nurseries, and for reproduction [1]–[6]. Although there has been a long-standing debate on the role of salt marsh detritus in the direct support of higher trophic levels [7]–[11], several studies have linked access to invertebrate prey on the marsh surface to measurable changes in fish growth, weight gain, and energy storage [4], [12]–[16]. High quality salt marsh habitat facilitates secondary production in coastal waters as nekton are consumed by higher trophic levels [17]–[19].

Throughout the United States, >50% of tidal salt marshes have decreased in size and quality [20] because of disturbances such as interstate commerce, urban and shoreline development, and livestock rearing [21], [22]. Roads, bridges, and dikes constructed through salt marshes restrict tidal flow when associated culverts are undersized, resulting in marsh compaction and subsidence through the loss of inorganic sediments from tidal deposition and the oxidation and decay of drained peat deposits [23]. Tidal restrictions also facilitate plant invasions and further degrade habitat quality for resident nekton species [24], [25].

Introduced *Phragmites australis* subsp. *australis* (hereafter, “introduced *P. australis*”) has widely invaded oligohaline to polyhaline salt marshes throughout the mid-Atlantic and New England regions of North America [26]–[28]. This invasive macrophyte takes advantage of reduced salinity and increased disturbance and nitrogen availability behind tidal restrictions and forms near monocultures that decrease native plant diversity, temperature, and light [29], [30]. The dense belowground network of introduced *P. australis* roots and rhizomes and high aboveground biomass mat of living and slowly decomposing organic matter [29] that traps mineral and organic sediment can counteract the effects of marsh subsidence by raising marsh surface elevation. However, high rates of marsh accretion (3–4 mm per year) [31] can elevate the marsh platform to the extent that daily high tides may no longer flood the marsh surface [22]. In addition, during the later stages of *P. australis* invasion small water-filled marsh pools and depressions are often reduced [32], [33]. Restoration of tidal flow into restricted marshes has successfully decreased the cover of this invader [24], [30], [34], [35] and restored ecological function for multiple taxa [25], [36], [37].

Previous studies in New England have used measures of faunal presence/absence, quantity, richness, and diversity to assess habitat quality in tidally restricted marshes invaded by *P. australis* and tidally restored marshes relative to reference (*Spartina alterniflora*) marshes. Decreases in bird species richness, density, and abundance were documented in restricted marshes [36], [38], but nekton response was variable across studies, with density, abundance, and species richness varying by site and species [25], [36], [37], [39], [40]. Tidally restored sites exhibit wide variation in support of nekton for several years post-restoration while hydrologic, environmental, and physical variables respond over time to increased tidal flooding and duration [25], [36], [41], [42]. Raposa and Talley [43] suggest the variability in restoration response may be related to whether the marsh was previously diked/drained or diked/impounded, with the former showing increased nekton density and the latter showing decreased nekton density post-restoration.

Several studies have acknowledged the need to move beyond the collection of community data (e.g., density, richness) to assess the functional response of nekton to tidal restrictions and restoration [16], [43], [44]. Fish condition and growth are affected by habitat characteristics (e.g., prey availability, predation, competition, water quality, parasite presence, etc.) and by the physiology of the fish species (e.g., reproductive status, life history stage, sex, etc.) [45]–[48]. Fish exhibit life-long tradeoffs in resource allocation to metabolism, somatic growth, reproduction, and energy (lipid) storage [49], [50], with the latter essential to their ability to cope with environmental stress and successfully overwinter in northern climates [49], [51]. Resident salt marsh fish such as the mummichog, *Fundulus heteroclitus,* gain a significant portion of their energy by foraging on the marsh surface at high tide but show significant decreases in growth rate and weight gain when they only have access to unvegetated creek beds and pools [4], [12], [14]. Therefore, a decrease in marsh surface access or habitat quality resulting from tidal restrictions and *P. australis* invasion may result in detectable tradeoffs to fish condition, growth, and ultimately, survival.

Morphological and physiological indicators have been used to examine habitat quality for fish residing in different environments [5], [16], [46], [48], [52]–[54]. At the morphological level, the relationship between fish length and wet weight using regression and indices such as the Fulton’s Condition Factor (K) can be used to infer the well being of fish and are based on the premise that heavier fish of a given length are in better condition [55]. At the biochemical level the analysis of proximate body composition (% lipid, % lean dry mass, % water) is used to estimate resource allocation to energy storage vs. body structure [49], [52]. Habitat quality influences fish growth rate; therefore, if a linear relationship exists between fish size and otolith size [56], the mean daily width of the marginal otolith increments can be used as an index of recent daily growth [48], [57], [58]. Further, age class distributions using the annuli of otoliths and scales provide information on habitat suitability for different life history stages [54], [59]. Parasite prevalence and infection intensity have been used as indicators of environmental quality; however, the responses of parasite communities and their hosts vary depending on exposure time, parasite life cycle (direct or indirect), and environmental perturbations present (e.g., sewage, eutrophication, pollution, thermal stress, etc.) [53], [60]–[62]. Nonetheless, parasites are energetically costly and infection may result in tradeoffs to lipid storage, reproduction, and growth [16], [61].

Our study builds on earlier work by directly linking habitat quality to measurable attributes of fish health and productivity. We examined the influence of habitat quality on fish condition and growth using the above morphological and physiological indicators in order to address the following research questions: 1) Does the condition and growth of fish residing in tidally restricted marshes invaded by *P. australis* differ from fish in unrestricted, uninvaded (reference) marshes? 2) Can we detect a difference in the condition and growth of fish residing in reference marshes vs. those that have been tidally restored to remove *P. australis*? 3) Are differences in fish condition and growth between the restricted, restored, and reference marshes consistent across regions, seasons, and for both males and females?

## Methods

### Ethics Statements

Our study was carried out in strict accordance with the American Veterinary Medical Association Guidelines on Euthanasia and was approved by the University of Rhode Island Institutional Animal Care and Use Committee (protocol #AN09-05-020). Permission for collections were given by the Connecticut Department of Environmental Protection (#SC-10021), Rhode Island Department of Environmental Management (#2010-39), Massachusetts Division of Marine Fisheries (#159948), National Park Service Cape Cod National Seashore (#CACO-2010-SCI-0016), Rachel Carson National Wildlife Refuge (#53553-2009-05, 2010-05, 2011-10), and Maine Department of Marine Resources (#2009-53-00, 2010-60-01, 2011-45-02).

### Study Sites and Sampling Locations

**Figure 1:**
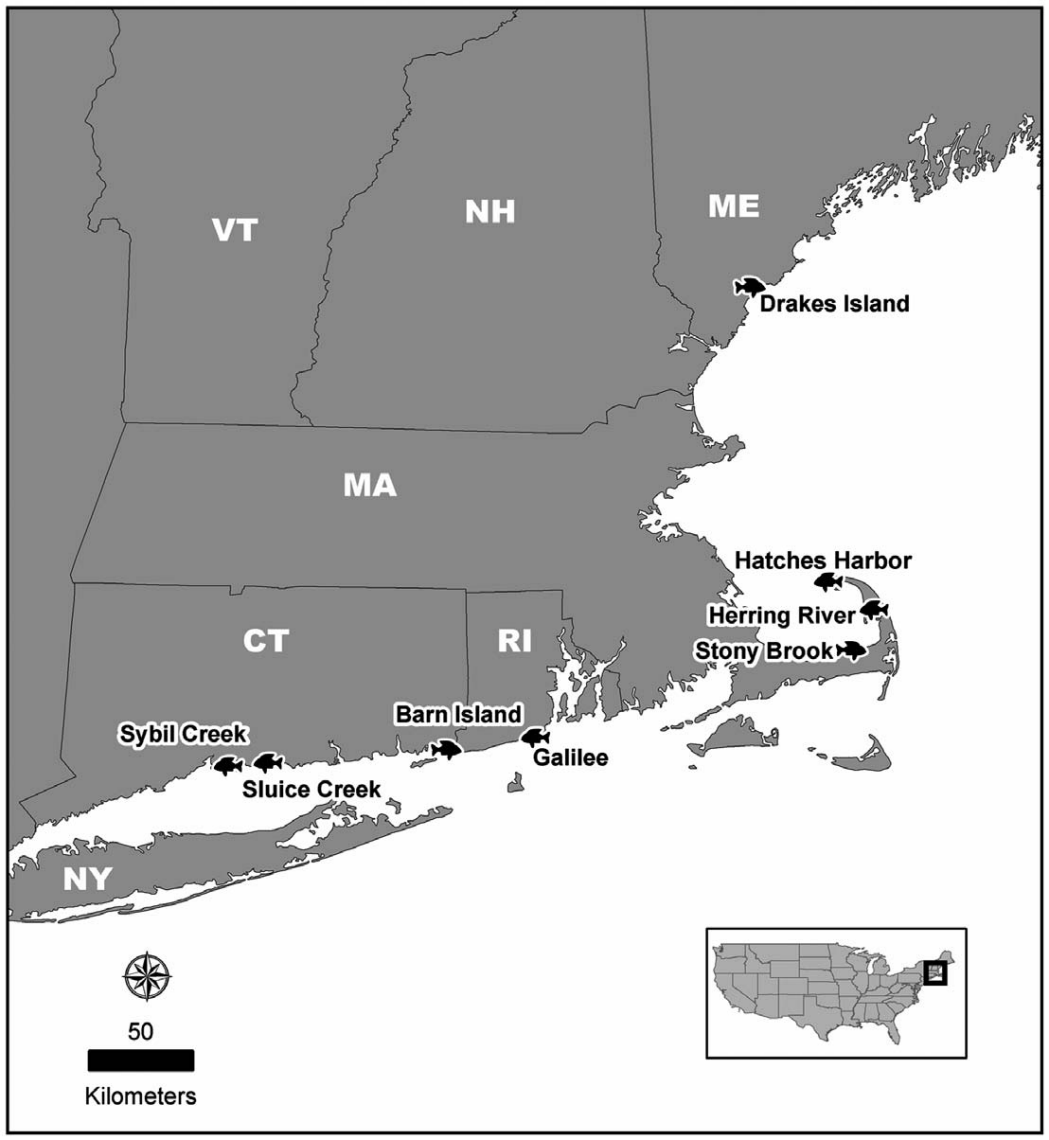
Map of study site locations in New England.

We selected four tidally restricted (hereafter, “restricted”) and four tidally restored (“restored”) salt marshes invaded by introduced *P. australis* in New England spanning Connecticut to Maine (Fig. 1, Table 1). Each restricted or restored site was paired with an adjacent downstream, unrestricted (“reference”) site that was sampled on the same day (n = 16 marshes; 4 restricted, 4 restored, 8 reference). Three sampling stations were randomly selected *a priori* along the main tidal creek within each of the 16 marshes (n = 48 experimental units). Because we employed a matched pairs experimental design, data from restricted marshes were only compared to data from the adjacent reference marshes, and data from restored marshes were only compared to data from adjacent reference marshes (i.e., four “marsh types” were analyzed; restricted/reference; restored/reference; Table 1). At the Stony Brook, MA site two undersized, failing culverts were replaced between the first and second year of our study due to funds appropriated for ‘shovel-ready’ habitat restoration projects (American Recovery and Reinvestment Act of 2009). However, because the other sites had tidal restrictions removed 11–22 years earlier we still classified year 2 data as restricted in the analysis.

**Table 1:** Characteristics of our New England study sites.^a^

Hydrologic Status/Marsh Type	Site	Latitude/Longitude	Region	System Type	Purpose ofRestriction	YearRestricted	YearRestored	Marsh Size(ha)	Tidal Range(m)	Salinity (ppt)
Restored	Barn Island (IP3),Stonington, CT	41°20′22′′N, 71°52′29′′W	LIS	marsh meadow	waterfowl/hunting	1947	1991	11	0.87	23.42
Restored	Drakes Island, Wells, ME	43°19′49′′N, 70°33′30′′W	GOM	marsh meadow	agriculture	1848	1988/2005^b^	35	0.86	29.54
Restored	Galilee, Galilee, RI	41°22′42′′N, 71°30′08′′W	LIS	marsh meadow	travel/commerce	1956	1997	40	0.47	30.84
Restored	Hatches Harbor,Provincetown, MA	42°03′56′′N, 70°14′09′′W	GOM	marsh meadow	mosquito/flood control	1930	1999	40	0.55	31.69
Reference (*restored*)	Barn Island (IP3),Stonington, CT	41°20′22′′N, 71°52′29′′W	LIS	marsh meadow	n/a	n/a	n/a	28	0.82	26.58
Reference (*restored*)	Drakes Island,Wells, ME	43°19′49′′N, 70°33′30′′W	GOM	marsh meadow	n/a	n/a	n/a	28	2.12	31.47
Reference (*restored*)	Galilee, Galilee, RI	41°22′42′′N, 71°30′08′′W	LIS	fringing marsh	n/a	n/a	n/a	11	0.59	30.73
Reference (*restored*)	Hatches Harbor,Provincetown, MA	42°03′56′′N, 70°14′09′′W	GOM	marsh meadow	n/a	n/a	n/a	34	0.65	31.70
Restricted	Herring River,Wellfleet, MA	41°55′54′′N, 70°03′49′′W	GOM	tidal riverine	travel/commerce	1908	n/a	42^c^	0.50	17.08
Restricted	Sluice Creek,Guilford, CT	41°16′32′′N, 72°39′52′′W	LIS	tidal riverine	agriculture	1847	n/a	31	0.70	16.40
Restricted	Stony Brook,Brewster, MA	41°45′05′′N, 70°06′49′′W	GOM	tidal riverine	agriculture/salt works	1700′s	2011^d^	8	0.60	11.13
Restricted	Sybil Creek,Branford, CT	41°15′43′′N, 72°47′59′′W	LIS	tidal riverine	flood control	early 1900′s	n/a	30	0.36	20.73
Reference (*restricted*)	Herring River,Wellfleet, MA	41°55′54′′N, 70°03′49′′W	GOM	fringing marsh	n/a	n/a	n/a	40	2.30	28.89
Reference (*restricted*)	Sluice Creek,Guilford, CT	41°16′32′′N, 72°39′52′′W	LIS	marsh meadow	n/a	n/a	n/a	29	1.70	22.36
Reference (*restricted*)	Stony Brook,Brewster, MA	41°45′05′′N, 70°06′49′′W	GOM	marsh meadow	n/a	n/a	n/a	36	1.40	30.80
Reference (*restricted*)	Sybil Creek,Branford, CT	41°15′43′′N, 72°47′59′′W	LIS	marsh meadow	n/a	n/a	n/a	14	1.80	22.14

a Citations: [23,25,35,63–76; Dr. Michele Dionne, Wells NERR, unpublished data].

b Drakes Island (restored): Unplanned partial restoration in 1988 (flapper gate fell off during storm); self-regulating tide gate installed in 2005.

c Herring River (restricted): Total suitable habitat area for my study (upstream of Chequessett Marsh Rd., downstream of High Toss Rd); total area for potential restoration- 445 ha.

d Stony Brook (restricted): Two failing culverts were replaced between year 1 and year 2 of my study (winter 2010–2011).

Site characteristics are reviewed in Table 1 [63]–[76]. Introduced *P. australis* was more prevalent in restricted marshes than in the restored marshes (K.L. Dibble, personal observation). At restored marshes the increase in tidal flow and associated salinity over time has decreased the cover of introduced *P. australis* and/or forced distributional shifts of the invasive plant toward the upland edge of the marsh [35], [71], [75]. The restored marshes are all marsh meadow systems with restrictions dating back to 1848 that were put in place to enhance hunting, agriculture, commerce, and flood control. They have been undergoing restoration for 1–2 decades as evidenced by similarity in mean tidal range and salinity relative to adjacent reference marshes. The restricted marshes are all tidal riverine systems diked dating back to the 1700’s for agriculture (salt hay farming), salt works, flood control, and/or to facilitate commerce/travel [63]–[76]. Mean tidal range and salinity in the restricted marshes is lower relative to adjacent reference marsh meadows and fringing marshes (Table 1), facilitating the observed invasion by *P. australis*. Although our study design does not allow us to separate the effects of tidal restrictions from the effects of *P. australis* invasion, these two factors are often successive in New England salt marshes and both work to reduce tidal range and marsh surface access and hence, nekton support functions [24], [40], [71], [77].

### Field Data

We collected data on water column salinity (ppt), temperature (°C), and dissolved oxygen (mg/L) at each station using a YSI-85 (2010) and a YSI Pro-2030 (2011). Water quality data were spot measurements (n = 1 per station per time period) taken from approximately mid-way through flood tide to peak high tide (prior to ebbing) when fish were removed from the water column. We collected water quality data from all sites in fall 2010, summer 2011, and fall 2011, but only from the four southern sites in Connecticut and Rhode Island in summer 2010 (due to equipment malfunction). Sampling dates were as follows: summer 2010 (7/12–7/25, 7/29), fall 2010 (9/22–10/3), summer 2011 (7/11–7/23), and fall 2011 (9/25–10/7). Study sites were sampled along a south-to-north transect in summer, and then along a north-to-south transect in fall to account for seasonality changes in the marshes. For gravidity data, sites were sampled during one lunar cycle in summer 2010 (new moon on 7/11/10, full moon on 7/26/10), while sites were sampled during the days leading up to and just past full moon (7/15/11) in summer 2011.

On flood tide at each station on every sample date we deployed two minnow traps containing bait in enclosed mesh packets (to prevent consumption). All traps were placed within one meter of the salt marsh bank parallel to the shore in the main tidal creek of each system [78]. After 30 minutes we combined the fish contents from both traps and randomly selected eight male and eight female adult *F. heteroclitus* (>40 mm in fork length) representing the longest (2 male, 2 female), shortest (2 M, 2 F), and intermediate (4 M, 4 F) size ranges of fish available. Sixteen fish were selected per station (15 fish analyzed, 1 stored in −80°C freezer) because previous power analyses and other analyses using nekton species composition, abundance, length, and biochemical data indicated that replicate samples of 5–15 *F. heteroclitus* was sufficient to detect trends between marsh types [16], [79], [80]. We measured fork length (nearest millimeter) and wet weight (nearest centigram), quantified external parasites (ectoparasites) on the body surface, and then humanely euthanized fish in the field using a sharp knife and the guillotine method. In the laboratory, we quantified internal parasites (endoparasites) infecting the liver, heart, and abdominal cavity, recorded female gravidity status (eggs present/absent), and removed and discarded fish digestive tracts and regurgitated food. We calculated parasite infection intensity, prevalence, and weighted prevalence [81], [82] using all data from 2010–2011. We rinsed fish in DI water then froze and freeze-dried the 16 fish from each station. Of the 16 fish, five female and five male fish were randomly selected, ground, and stored in a −80°C freezer for lipid extraction. Five fish (2 male/3 females, or 3 female/2 males) from each station were stored in a −80°C freezer, with the field-decapitated head used for otolith measurements.

### Laboratory Data

#### Proximate body composition (lipid/lean dry/water)

In 2010 and 2011 we extracted whole-body lipid reserves from 1,920 adult fish (n = 960 fish/year). Powdered fish samples were packed into pre-weighed Whatman cellulose extraction thimbles, dried to a constant weight in a 50°C oven overnight, re-weighed pre-extraction, extracted for six hours using petroleum ether and a Soxhlet apparatus, dried in a 50°C oven overnight, and then re-weighed post-extraction [83]. We selected petroleum ether as the non-polar lipid solvent because it is highly effective at removing neutral lipids (energy reserves) while minimizing loss of non-lipid, structural material [83]. We determined the percent lipid (% dry), lean dry mass (% dry), and water (% wet) of individual fish using the following equations:
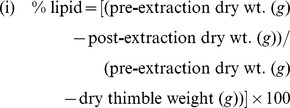


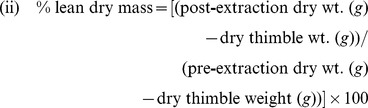


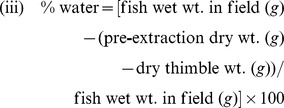



#### Fish age and recent daily growth rate

Radtke and Dean [84] verified daily increment formation using *F. heteroclitus* sagittae, finding that daily increments form regardless of growth rate, which is faster at higher water temperatures (30°C vs. 24°C). Therefore, we can use sagittal otolith increments to determine the age and growth rates of *F. heteroclitus* living in different environmental conditions. We removed pairs of sagittal otoliths from 960 adult fish (n = 480 fish/year) using a dissecting microscope and the ‘crunch and crumble’ extraction method [85]. Otoliths were cleaned in distilled water and 10% bleach, treated with 95% ethanol, and then dried in an oven (1 h at 50°C). We mounted the pair of otoliths on standard microscope slides (sulcus side down), covered in Cargille immersion oil (Type FF, nonfluorescing). All measurements were done using the right otolith for consistency, unless the right was broken or could not be located during extraction. In that case, measurements were done on the left otolith. Using a Zeiss Stereo Microscope (Discovery, v12), high-powered objective (Plan Apo S 3,5x), and image analysis software (AxioVisionRel.4.8), we recorded fish age under transmitted light (# dark annular rings, magnification 100x) [59].

To verify the relationship between otolith growth and somatic growth [56], we took three measurements of total otolith radius (µm) and calculated the mean. We also took three measurements of total otolith height and length and calculated the mean for each otolith. Under reflected light and high magnification (560x), we measured the distance between the margin of the otolith in the postero-dorsal region [86], [87] back to the 10^th^ daily growth ring three times, took the mean of the three separate measurements, and divided the measurement by 10 days to compute the Recent Growth Index (in µm) [48], [57]. Recent daily growth measurements from readable otoliths were re-measured by a second reader 2–3 months later. We discarded any otoliths for which the first and second growth measurements were not within 10% of each other and report the mean of the first and second measurements [88]–[91].

#### Fulton’s K and length-weight relationships

During field collections, we recorded the fork length and wet weight of 1,487 fish in 2010 and 1,529 fish in 2011. We use a common morphometric index of fish condition, Fulton’s Condition Factor (K), to compare the condition of adult fish. It is calculated using the following equation:
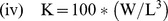
Where W = weight of fish (mg) and L = fork length of fish (mm) [55]. Fulton’s K assumes that heavier fish of a given length are in better condition; therefore this index can be used as an indicator of energy storage. We compared the results of K to the results of Multiple Linear Regression using categorical variables for each marsh type (restricted, restored, reference).

### Statistical Analyses

In total, our main experiment included two paired marsh comparisons (restricted vs. reference; restored vs. reference). Each of the 48 Experimental Units (EU) were visited twice in 2010 (n = 96) and twice in 2011 (n = 96). Because we collected samples from each EU over time, we analyzed data using repeated measures mixed model ANOVA (Statistical Package SAS, v 9.2). To avoid pseudoreplication we took the mean of each response variable collected on each EU on each sampling date (i.e., the mean of 10 fish for proximate body composition, 5 for recent daily growth, 16 for morphology). The exception to this was water quality data, for which we had one data point per EU on each sample date (except the four sites in summer 2010, as discussed above). We used SLICES in the model to examine interaction effects to determine whether there were significant differences in the response after explanatory variables were incorporated into the model (i.e., marsh type, time, region, parasitism status, gravidity, sex). We used Heterogeneous Autoregressive (1) as our covariance structure because it assumes that data that are farther apart in time will be less similar and that each time period has its own unique variance. Assumptions of normality and equality of variances within datasets were verified prior to all statistical analyses. We arcsine-square-root transformed our percent lipid, lean dry, and water data prior to analysis. For proximate body composition and growth rate data we incorporated mean fish length into the model as a covariate to ensure significant differences were attributable to marsh type and not differences in fish size [49]. Significance was determined at the α = 0.05 level. We developed figures and graphics using SigmaPlot (v. 9.0) and the R statistical software environment (v. 2.14.1).

Proportions of gravid and/or parasitized fish were compared between habitats using Two Sample Tests for Proportions; data is reported as the mean ± proportional standard deviation. A continuity correction was conducted for the restricted vs. reference gravidity data to increase the quality of the normal approximation to the binomial distribution. To determine whether it was necessary to remove afflicted individuals from the analysis, we quantified the effects of parasitism and gravidity on fish lipid mass and morphology using repeated measures ANOVA. Due to unequal sample size (>2×), we analyzed the effects of parasitism/gravidity on recent daily growth using Welch’s t-tests. We used Simple Linear Regression to model the relationship between fish length and otolith radius in healthy fish (i.e., those without ecto/endoparasites or eggs present) and examined homogeneity of fish age class distributions using Chi Square Tests of Homogeneity. Lipid and lean dry mass results are presented as a percentage of fish dry weight, water mass as a percent of wet weight, growth as the mean recent daily growth increment of the otolith (in micrometers), and morphology as a unitless index value (K). Means are reported for each statistic ± standard deviation.

## Results

### Field Data

#### Water quality

We collected 164 sets of water quality data from the 48 stations from 2010–2011 (Table 2). In the restored vs. reference sites in Long Island Sound (LIS), we found no significant difference in salinity (p = 0.9717; t_40_ = 0.04), temperature (p = 0.4287; t_40_ = −0.80), or dissolved oxygen (p = 0.3747; t_40_ = −0.90), which mirrored results in the Gulf of Maine (GOM; salinity: p = 0.9542, t_40_ = −0.06; temperature: p = 0.8690, t_40_ = −0.17; dissolved oxygen: p = 0.5496, t_40_ = 0.60). In LIS, we found a highly significant difference in salinity between restricted vs. reference sites (p = 0.0019; t_40_ = 3.33), but not for temperature (p = 0.1588; t_40_ = −1.44) or dissolved oxygen (p = 0.3821; t_40_ = −0.88), which also mirrored results in the GOM (salinity: p<0.0001, t_40_ = 11.89; temperature: p = 0.1409, t_40_ = −1.50; dissolved oxygen: p = 0.2253, t_40_ = −1.23; Table 2).

**Table 2:** Mean water quality 2010–2011, by marsh type (standard deviations in parentheses; data pooled across regions and seasons).

Response	Salinity (ppt)	Temperature (°C)	Dissolved Oxygen (mg/L)	N
Restored	28.62 (6.79)	21.44 (3.62)	6.98 (2.78)	42
Reference (*restored*)	29.89 (3.65)	20.41 (3.25)	7.15 (2.34)	41
Restricted	14.19 (9.65)	21.98 (3.92)	7.50 (2.44)	39
Reference (*restricted*)	25.50 (4.78)	21.17 (3.86)	6.44 (2.55)	42

#### Parasitism and gravidity

**Figure 2:**
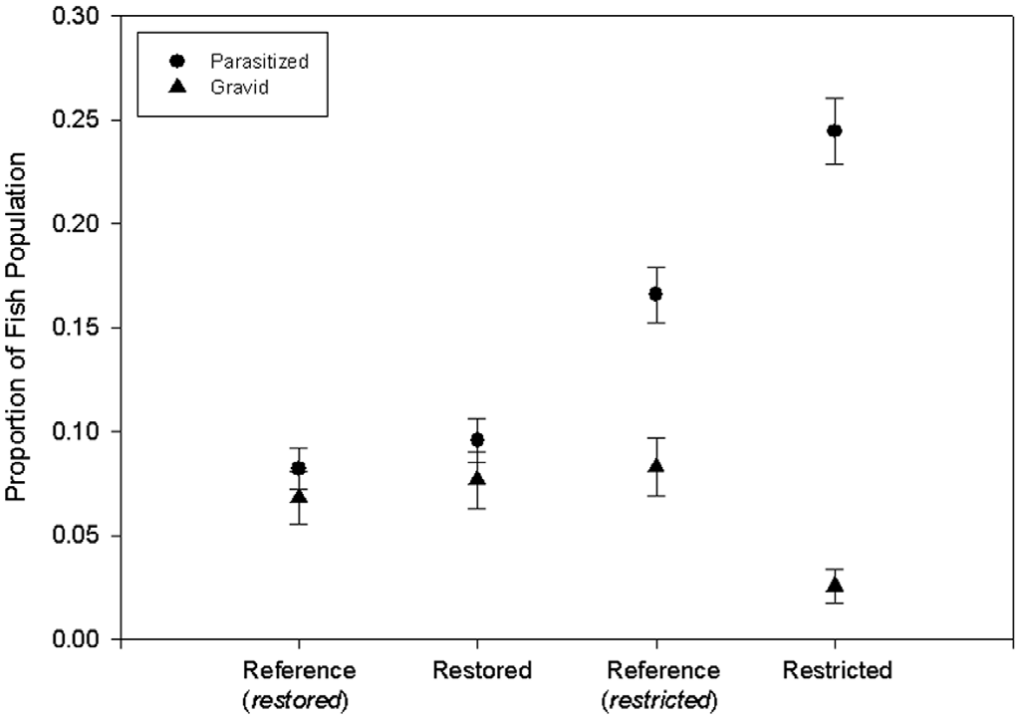
Proportion of fish parasitized (circles; females and males) or gravid (triangles; females only) by marsh type. Data is presented as the mean proportion ± standard deviation.

*Fundulus heteroclitus* were infected by a range of parasites including sea lice (Branchiura), anchor worms (Copepoda), flat worms (Monogenea, Digenea), internal cavity worms infecting the liver, intestines, and mesenteries (Cestoda, Acanthocephala), and the internal nematode parasite, *Eustrongylides* spp. We grouped data by parasite location (ecto/endo) and found that fish in the restricted marshes had the highest overall prevalence and weighted prevalence of parasite infection among the marsh types (Table 3). Overall infection intensity was also highest for the restricted marsh fish. We analyzed the proportion (prevalence) of parasitized fish by marsh type and found no significant difference between the reference (n = 62/755; 8.21±1.00%) vs. restored marsh fish (n = 72/751; 9.59±1.07%; p = 0.3486, Z = −0.94; Fig. 2; Table 3). However, we found significantly more parasitized fish in restricted marshes (n = 185/756; 24.47±1.56%) in comparison to adjacent reference marshes (n = 125/754; 16.58±1.35%; p = 0.0001; Z = −3.80; Fig. 2; Table 3). Within the female population collected over our entire study period (2010–2011), there was no difference in the proportion of gravid fish in the reference (n = 27/397; 6.80±1.26%) vs. restored marshes (n = 29/378; 7.67±1.37%; p = 0.6397; Z = −0.47; Fig. 2). However, we did find significantly fewer gravid fish inhabiting the restricted (n = 10/392; 2.55±0.80%) vs. reference marshes (n = 32/385; 8.31±1.41%; p = 0.0007; Z = 3.55; Fig. 2) from 2010–2011.

**Table 3:** Parasites infecting *Fundulus heteroclitus* by marsh type, 2010–2011.

	Ectoparasites	Endoparasites	Total
Restored			
Abundance	68	29	97
Total Infected	56	18	72
Infection Intensity	1.21	1.61	1.35
Prevalence	7.46%	2.40%	9.59%
Weighted Prevalence	9.05%	3.86%	12.92%
Reference (*restored*)			
Abundance	62	42	104
Total Infected	53	13	62
Infection Intensity	1.17	3.23	1.68
Prevalence	7.02%	1.72%	8.21%
Weighted Prevalence	8.21%	5.56%	13.77%
Restricted			
Abundance	91	396	487
Total Infected	77	132	185
Infection Intensity	1.18	3.00	2.63
Prevalence	10.19%	17.46%	24.47%
Weighted Prevalence	12.04%	52.38%	64.42%
Reference (*restricted*)			
Abundance	83	195	278
Total Infected	69	70	125
Infection Intensity	1.20	2.79	2.22
Prevalence	9.15%	9.28%	16.58%
Weighted Prevalence	11.01%	25.86%	36.87%

### Laboratory Data

#### Proximate body composition (lipid/lean dry/water)

We successfully extracted whole body lipids from 1,915 of 1,920 fish captured from 2010–2011. Approximately 14.67% (n = 281) of the fish analyzed for proximate body composition were parasitized. Incorporation of parasitism status into a repeated measures ANOVA revealed a significant negative effect on lipid stores when fish length was added as a covariate (p = 0.0181; F_1,37_ = 6.12), with lower lipid reserves in parasitized fish (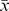 = 7.90±2.89%) than in unparasitized fish (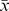 = 8.44±2.55%). Approximately 6.84% (n = 68) of the fish analyzed for proximate body composition were gravid. The unparasitized gravid female fish had significantly less lipid than the non-gravid females (p<0.0001; F_1,20_ = 88.44). These effects were highly significant and consistent across marsh types, with gravid females averaging 4.89±1.92% lipid and non-gravid females averaging 8.33±2.04% lipid, indicating a significant allocation of energy reserves to reproduction. Since we found significant negative effects of parasitism and gravidity on lipid mass, we removed all gravid and afflicted fish from further analyses to eliminate confounding effects and clarify the interpretation of our results (n = 338/1,915 removed; 17.65%). The fish in all subsequent lipid analyses represent unparasitized, non-gravid (termed “healthy”) individuals in the population (n = 1,577). A consequence, however, is that the mean from each EU became unbalanced (i.e., n<10).

**Figure 3:**
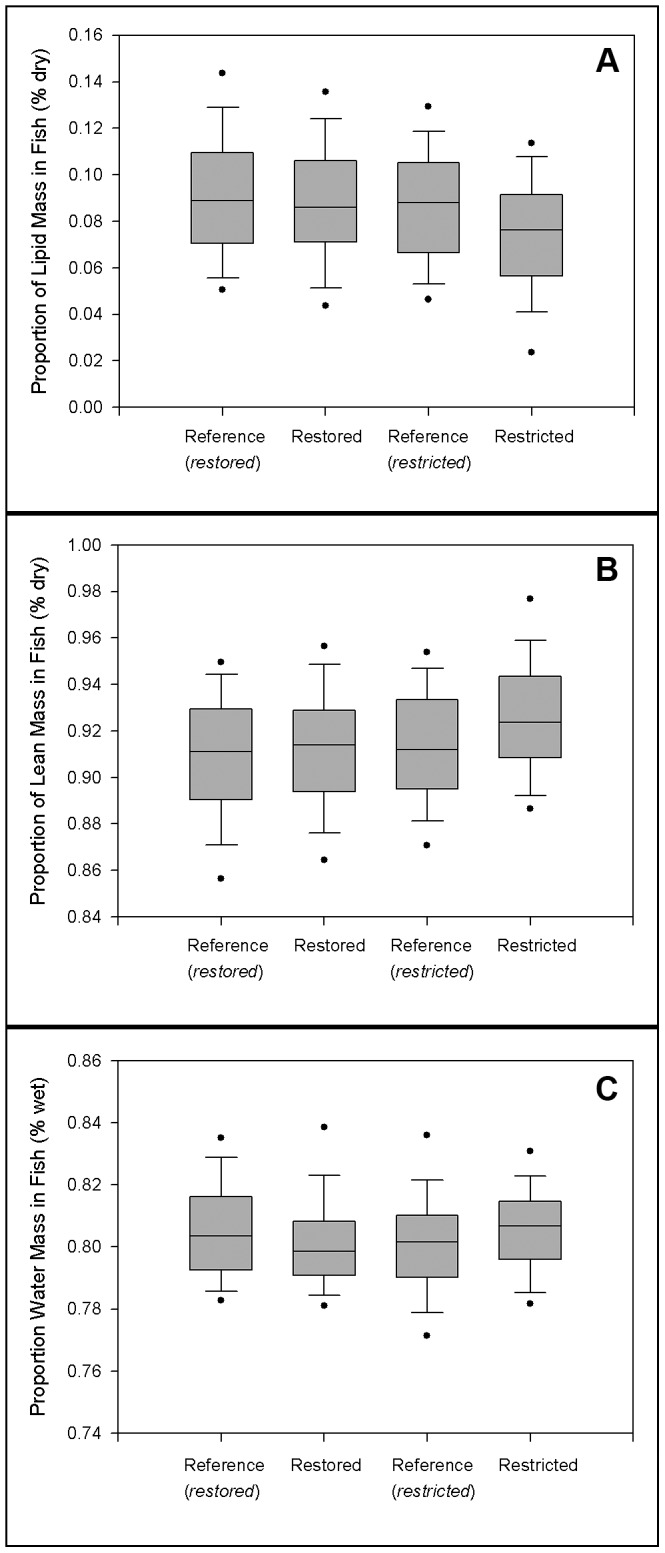
Proximate body composition of fish. Healthy fish only- data pooled across seasons, regions, and sex. Outlier circles represent the 5^th^ and 95^th^ percentiles and error bars the 10^th^ and 90^th^ percentiles for each population. (A) % lipid mass (dry weight). (B) % lean mass (dry weight). (C) % water mass (wet weight).

Using pooled data by sex across habitat/time periods, we found that fish in the Gulf of Maine had significantly more lipid than those in Long Island Sound (p<0.0001; F_1,40_ = 125.70), which was consistent by season and suggests influences of countergradient variation [92], [93]. Overall, females contained more lipid than males (p = 0.0001; F_1,40_ = 18.64; Table 4). We found significant differences overall by season (p<0.0001; F_3,120_ = 30.67), with fall fish (pre-hibernation) having significantly more lipid than summer fish (post-reproduction) in both 2010 (p = 0.0008; t_120_ = 3.43) and 2011 (p<0.0001; t_120_ = 7.66; Table 4). By marsh type, we found no difference in the lipid mass of healthy fish inhabiting the restored vs. reference marshes (p = 0.2445; t_40_ = 1.18; Table 4; Fig. 3a). When we analyzed the interactions between marsh type, region, time, and sex we found a significant difference between the restored and reference habitats in LIS (p = 0.0278; t_40_ = 2.28), which was likely driven by differences in males in fall 2010 (p = 0.0129; t_40_ = 2.60). We found a highly significant difference in lipid mass between fish inhabiting the restricted vs. reference marshes (p = 0.0013; t_40_ = 3.45; Tables 4–5; Fig. 3a). Significant differences between restricted and reference marsh fish held with comparisons of fish from the GOM, LIS, in three of the four time periods sampled, and for both males and females (Table 5).

**Table 4:** Mean proximate body composition of fish in study, 2010–2011 (standard deviations in parentheses; data by marsh type are pooled across regions, seasons, and sex; data by region, season, and sex are pooled across marsh types; reference marshes adjacent to the restored and restricted marshes are noted in parentheses).

Response	Lipid(% of dry)	Total lipid(g)	Lean mass(% of dry)	Total leanmass (g)	Water(% of wet)	Totalwater (g)	Fish length(mm)
Restored	8.78 (2.69)	0.08 (0.05)	91.22 (2.69)	0.84 (0.38)	80.14 (1.66)	3.62 (1.50)	69.7 (9.5)
Reference (*restored*)	9.09 (2.63)	0.06 (0.04)	90.91 (2.63)	0.63 (0.32)	80.56 (1.62)	2.81 (1.38)	63.6 (9.4)
Restricted	7.48 (2.61)	0.06 (0.04)	92.52 (2.61)	0.75 (0.32)	80.54 (1.42)	3.26 (1.29)	67.0 (8.7)
Reference (*restricted*)	8.62 (2.49)	0.10 (0.07)	91.38 (2.49)	0.96 (0.46)	80.04 (1.75)	4.59 (5.21)	71.6 (10.0)
Gulf of Maine	9.90 (2.20)	0.10 (0.06)	90.10 (2.20)	0.92 (0.44)	80.20 (1.58)	4.23 (3.89)	71.2 (10.4)
Long Island Sound	7.08 (2.33)	0.05 (0.02)	92.92 (2.33)	0.67 (0.29)	80.44 (1.67)	2.90 (1.13)	64.7 (8.0)
Summer 2010	7.51 (2.22)	0.06 (0.04)	92.49 (2.22)	0.76 (0.28)	81.71 (1.54)	3.63 (1.25)	69.9 (7.1)
Fall 2010	8.41 (2.50)	0.08 (0.06)	91.59 (2.50)	0.85 (0.44)	80.05 (1.51)	3.60 (1.69)	69.7 (10.7)
Summer 2011	7.72 (2.25)	0.07 (0.05)	92.28 (2.25)	0.86 (0.41)	80.30 (1.10)	3.72 (1.67)	69.0 (9.4)
Fall 2011	10.31 (2.74)	0.08 (0.06)	89.69 (2.74)	0.71 (0.41)	79.24 (1.28)	3.33 (5.24)	63.3 (10.3)
Males	8.23 (2.87)	0.07 (0.05)	91.77 (2.87)	0.75 (0.38)	80.18 (1.76)	3.24 (1.46)	66.9 (9.5)
Females	8.75 (2.42)	0.08 (0.05)	91.25 (2.42)	0.83 (0.41)	80.46 (1.48)	3.89 (3.86)	69.0 (10.1)

**Table 5:** Results of repeated measures ANOVA for the restricted vs. reference systems [Model terms: Marsh type (termed “Marsh”: comparison of restricted vs. reference); Time (comparison of the two marsh types within summer 2010, fall 2010, summer 2011, fall 2011); Region (comparison of the two marsh types within the Gulf of Maine vs. Long Island Sound)].

	% Lipid		% Lean Dry Mass		% Water		
Model Terms	Sign.	t-statistic	Sign.	t-statistic	Sign.	t-statistic	d.f.
Marsh	p = 0.0013	3.45	p = 0.0013	−3.45	p = 0.5213	−0.65	40
Marsh × Region							
GOM	p = 0.0116	2.65	p = 0.0116	−2.65	p = 0.3746	−0.90	40
LIS	p = 0.0305	2.24	p = 0.0305	−2.24	p = 0.9907	−0.01	40
Marsh × Time							
Summer 2010	p = 0.0519	1.96	p = 0.0519	−1.96	p = 0.4474	0.76	120
Fall 2010	p = 0.0112	2.58	p = 0.0112	−2.58	p = 0.3111	−1.02	120
Summer 2011	p = 0.0141	2.49	p = 0.0141	−2.49	p = 0.3092	−1.02	120
Fall 2011	p = 0.1970	1.30	p = 0.1970	−1.30	p = 0.5632	−0.58	120
Marsh × Sex							
Males	p = 0.0068	2.85	p = 0.0068	−2.85	p = 0.1892	−1.34	40
Females	p = 0.0027	3.20	p = 0.0027	−3.20	p = 0.7592	0.31	40
Marsh × Region × Sex							
GOM, Males	p = 0.0096	2.72	p = 0.0096	−2.72	p = 0.0400	−2.12	40
GOM, Females	p = 0.0801	1.80	p = 0.0801	−1.80	p = 0.4887	0.70	40
LIS, Males	p = 0.1964	1.31	p = 0.1964	−1.31	p = 0.8144	0.24	40
LIS, Females	p = 0.0088	2.75	p = 0.0088	−2.75	p = 0.7863	−0.27	40

We analyzed lipid-free dry mass (composed primarily of protein and bone/ash) in healthy fish to examine investment in body structure vs. lipid storage. Because we analyzed data on a dry weight basis, % lipid and % lean dry mass are the only two proportions in dry fish weight. Therefore, the statistics reported (Table 5) are nearly identical, but in the opposite direction. Overall, lean dry mass constituted a lower proportion of fish body weight in the GOM than in LIS (p<0.0001; F_1,40_ = 125.70) and lean dry mass in females was lower than that of males (p = 0.0001; F_1,40_ = 18.64; Table 4). By marsh type, we found no difference between the restored and reference sites in the proportion of mass allocated to structure (p = 0.2445; t_40_ = −1.18) or water (p = 0.6547; t_40_ = −0.45; Table 4; Figs. 3b,c). We found a highly significant difference between the restricted and reference sites in the proportion allocated to structural mass (p = 0.0013; t_40_ = −3.45) but not for water mass (p = 0.5213; t_40_ = −0.65; Tables 4–5; Figs. 3b,c). We also found no difference in water mass by region (p = 0.0826; F_1,40_ = 3.17) and for most of the interactions (Table 5).

#### Fish age and recent daily growth rate

**Figure 4:**
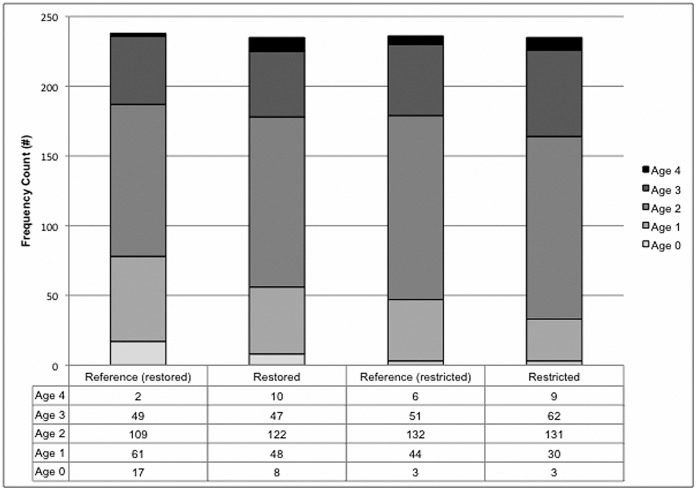
Number of fish captured by age group and marsh type.

Our capture and fish selection methodology was designed to gather information from a range of fish sizes present at each site, so we analyzed whether the proportion of age classes differed between marsh systems. We report age data from 465 fish in 2010 and 479 fish in 2011. From 2010–2011, we captured five age classes of fish (ages 0, 1, 2, 3, 4). Although it was not our intent to capture fish in the age 0 class (i.e., those in their first year of life), we captured 31 fish in fall 2011 that had grown to at least 40 mm and were therefore included in our field collections. Chi Square Tests of Homogeneity revealed a significant difference in age class distributions between restored vs. reference marsh systems (p = 0.0280; χ^2^_4_ = 10.8785; n = 473; Fig. 4) but not between the restricted vs. reference marshes (p = 0.3643; χ^2^_4_ = 4.3211; n = 471; Fig. 4). Within the four marsh types, the frequency of the smallest age classes (ages 0 and 1) was highest in reference marshes adjacent to restored sites (n = 78; 32.77%) and lowest in the tidally restricted marshes (n = 33; 14.04%). Fish in the other two marsh groups were intermediate (restored: 56 fish, 23.83%; reference adjacent to restricted: 47 fish 19.92%; Fig. 4). Therefore, reference marshes adjacent to restored marshes harbored the largest proportion of young fish.

Due to unclear daily growth rings or other structural abnormalities in the otoliths (e.g., irregular accretion of calcium carbonate along the edge, resulting in a scalloped morphology) we initially discarded 263 fish from our study, with an additional 155 discards due to a >10% difference between the first and second growth readings. In total we analyzed growth rate data from 542 fish from 2010–2011 (56.5%). Our approach is consistent with other studies that have selected only the clearest otoliths for microstructure analysis (top 15.7%) [91] or discarded data from up to 44.9% of samples due to imprecise increment patterns, accessory primordia, or errors during sample preparation [89], [92]–[94].

**Figure 5:**
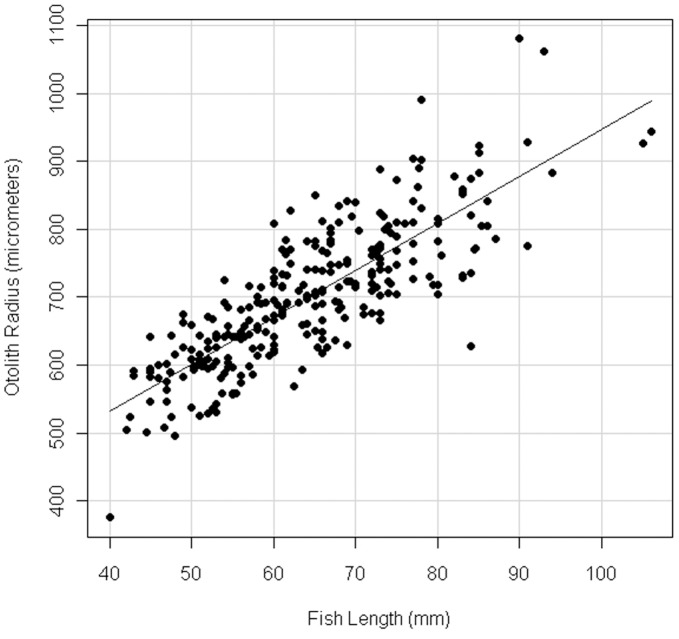
Fish length vs. otolith radius for healthy fish. (Otolith radius = −2.77341+0.09572*fish length; p<0.0001; r^2^ = 0.6628).

An analysis of the effects of parasitism and gravidity did not reveal significant negative effects on fish growth rate (p = 0.7739; t_94.26_ = −0.288); however, we removed an additional 81 parasitized and/or gravid individuals from the growth rate analysis to be consistent in our interpretation of results across physiological and morphological analyses, resulting in growth rate data for 461 healthy fish. Using simple linear regression we found a highly significant relationship between fish length and otolith radius for healthy fish (p<0.0001; r^2^ = 0.6628; Otolith radius = −2.77341 + 0.09572* fish length; Fig. 5). Therefore, the marginal ten increments of *F. heteroclitus* otoliths can be used as a reliable indicator of recent daily growth at our sites.

Using the healthy individuals in the population and fish length as a covariate, we found that females grow significantly faster than males (p = 0.0461; F_1,38_ = 4.25; Table 6), so we separated our model by sex. Unlike our lipid mass results, we found no difference in the growth rate of fish residing in the GOM vs. LIS (p = 0.2786; F_1,40_ = 1.21). However, we did find a significant effect of season in the marshes, with fish growing at a higher rate in summer than in fall in both 2010 (p<0.0001; t_93_ = −13.63) and 2011 (p<0.0001; t_93_ = −8.58; Table 6). The higher growth rate in 2010 across habitats, regions, and seasons corresponds generally to a lower investment in energy storage (Tables 4, 6), whereas in 2011 the relationship is reversed (lower growth rate, higher energy investment), indicating potential trade-offs in somatic investments that may shift from year to year. Seasonally, fish in the summer are growing faster but have depleted lipid stores, whereas in fall the fish are growing slower but have significantly higher lipid reserves.

**Table 6:** Mean otolith measurements for fish in study, 2010–2011 (standard deviations in parentheses; data by marsh type are pooled across regions, seasons, and sex; data for region, season, and sex are pooled across marsh types; reference marshes adjacent to the restored and restricted marshes are noted in parentheses).

Response	Daily Growth(µm)	Otolith Radius(µm)	Otolith Length(µm)	Otolith Height(µm)	Fish Length(mm)	Fish Wet Weight(g)
Restored	2.16 (0.66)	719.16 (102.21)	1496.63 (207.74)	1351.16 (157.28)	66.2 (11.9)	3.88 (2.20)
Reference (*restored*)	2.26 (0.74)	669.96 (99.81)	1393.41 (212.99)	1271.54 (167.68)	61.7 (11.5)	3.09 (1.84)
Restricted	2.21 (0.79)	692.70 (86.86)	1450.01 (193.28)	1324.26 (134.89)	61.2 (11.1)	2.98 (1.78)
Reference (*restricted*)	2.26 (0.75)	726.75 (107.94)	1559.24 (227.14)	1391.76 (158.14)	67.8 (12.7)	4.29 (2.79)
Gulf of Maine	2.20 (0.64)	681.37 (99.97)	1451.10 (229.76)	1317.49 (172.15)	67.0 (13.0)	4.05 (2.59)
Long Island Sound	2.24 (0.81)	721.39 (102.13)	1495.87 (209.91)	1348.28 (153.15)	62.0 (10.7)	3.18 (1.81)
Summer 2010	3.03 (0.64)	720.36 (92.25)	1501.03 (188.93)	1355.08 (134.94)	67.7 (10.0)	4.06 (1.96)
Fall 2010	2.09 (0.37)	719.18 (107.44)	1520.13 (250.34)	1362.19 (179.38)	66.0 (13.5)	3.91 (2.77)
Summer 2011	2.39 (0.54)	728.15 (103.99)	1521.30 (209.17)	1380.89 (153.29)	67.6 (10.6)	3.77 (1.85)
Fall 2011	1.53 (0.19)	655.91 (93.36)	1384.16 (202.22)	1261.84 (156.15)	58.4 (11.3)	2.84 (2.10)
Males	2.21 (0.71)	691.85 (96.71)	1461.51 (207.13)	1330.16 (157.45)	62.8 (10.8)	3.23 (1.72)
Females	2.23 (0.75)	712.53 (108.28)	1487.33 (233.71)	1336.65 (169.28)	66.1 (13.1)	3.99 (2.67)

By marsh type, we did not detect differences in the growth rate between fish residing in restored vs. reference marshes (p = 0.2506; t_40_ = 1.17), nor between fish in the restricted vs. reference marshes (p = 0.5153; t_40_ = 0.66; Table 6). However, we did detect a difference in growth rate between the restored and reference sites within the LIS region (p = 0.0389; t_40_ = 2.14) that mirrors our proximate body composition data. The difference in LIS appears to be driven by the males (p = 0.0201; t_38_ = 2.43) rather than the females, which were equivalent between marsh types (p = 0.5327; t_38_ = 0.63). For the restricted vs. reference fish, none of the interactions for growth rate by season, region, and time were significant (p>0.05), indicating that fish of similar lengths are growing at the same rate in both the restricted and reference habitats despite the differences in allocation of resources to lipid storage (Tables 4, 6).

#### Fulton’s condition factor (K)

Analysis of morphology data using Fulton’s K in our repeated measures ANOVA revealed no overall negative effect of parasitism/gravidity on fish condition (p = 0.7453; F_1,40_ = 0.11). However, to be consistent in our interpretation of results across analyses we removed all afflicted individuals from the analysis (n = 517). Using only healthy fish (n = 2,499), we found no significant difference between the restored vs. reference (p = 0.6273; t_40_ = −0.49) or the restricted vs. reference marsh fish (p = 0.4962; t_40_ = 0.69). Analysis of possible interactions between marsh type, region, time, and sex revealed only one significant difference between the reference and restricted marsh fish in fall 2010 (p = 0.0458; t_120_ = 2.02), with Fulton’s K indicating that reference marsh fish were in better condition than those in restricted marshes. We did find a difference between the summer and fall seasons in 2010 (p = 0.0016; t_120_ = −3.22) and a marginal difference in 2011 (p = 0.0570; t_120_ = −1.92), but the effect was in the opposite direction, with Fulton’s K labeling summer fish (post-reproduction) healthier than those in fall (pre-hibernation) in both years. In addition, this morphological index did not detect trends in condition between sexes (p = 0.3804; F_1,40_ = 0.89) or regions (p = 0.7849; F_1,40_ = −0.27) found using physiological indices.

#### Length-weight relationships

**Figure 6:**
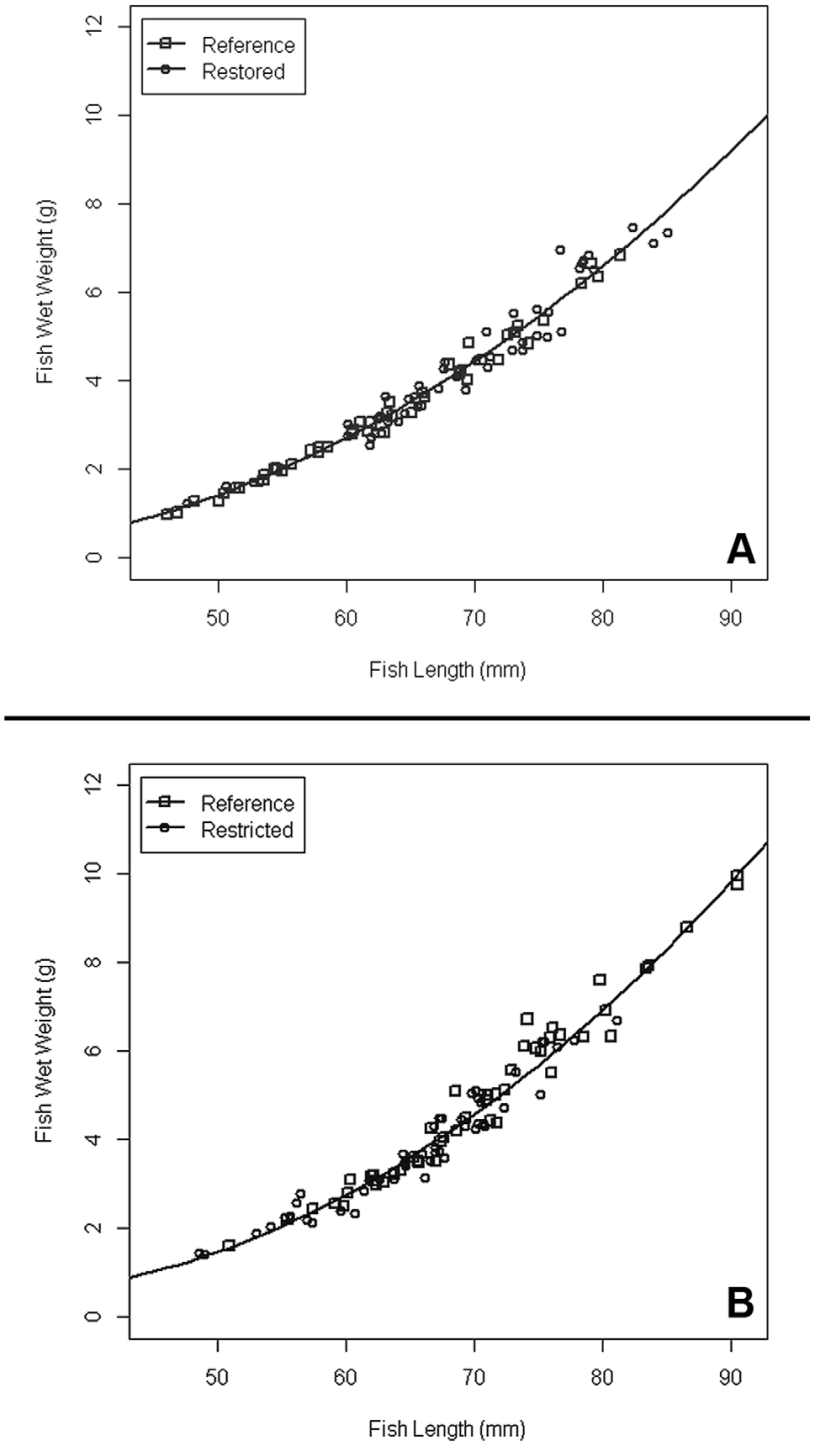
Fish length vs. wet weight for healthy fish. Data pooled across seasons, regions, and by gender. (A) Restored vs. reference fish. (B) Restricted vs. reference fish.

We analyzed length-weight relationships using Multiple Linear Regression (with categorical variables for the marsh types). Examination of fit statistics (AIC, AICC, BIC), output from the regression coefficient hypothesis tests, adjusted R^2^, and multicollinearity statistics (tolerance, variance inflation factor) revealed that quadratic models best explained the length-weight relationships for the restored, restricted, and reference marsh fish (Figs. 6a,b). The restored and reference fish populations were best explained by one line with the following equation (adj. R^2^ = 0.9691; p<0.0001; Fig. 6a):
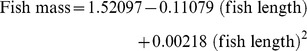



There was a strong positive linear relationship (p = 0.0058) as well as evidence of a curvilinear relationship (p<0.0001) between fish length and weight, with the intercept not significantly different from zero (p = 0.2329). For the restricted vs. reference marsh fish, one regression line again best explained both populations (adj. R^2^ = 0.9602; p<0.0001; Fig. 6b):
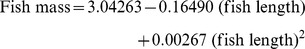



There was a strong positive linear relationship (p = 0.0009) and evidence of a curvilinear relationship (p<0.0001) between fish length and weight, with no difference in intercept (p = 0.0668). Combined with results using the Fulton’s K condition factor, results indicate that fish at our study sites are morphologically indistinguishable.

## Discussion

### Tradeoffs between Fish Growth, Energy Storage, and Reproduction

Our study demonstrates that fish residing in tidally restricted marshes invaded by *P. australis* allocate a greater proportion of resources to maintaining growth and body size than to building lipid stores relative to reference marsh fish. Results were consistent by gender, region, and for three of the four seasons sampled. Our findings suggest potential tradeoffs between growth, energy storage, and reproduction potentially due to reduced habitat quality, a decrease in access to invertebrate prey on the marsh surface, and lack of habitat refugia to avoid larger predators.

Access to the marsh surface is ultimately influenced by the frequency, depth, and duration of tidal flooding, with nekton exhibiting a positive relationship between marsh selection and flooding duration [6]. Although we did not collect data on marsh surface flooding at our sites, we collected samples on flood tide and only observed flooding of *P. australis* at the fringe of one of the four restricted sites (Herring River), whereas all reference and restored marshes flooded daily on high tides. Flooding of the marsh surface in invaded tidally restricted salt marshes is influenced by both the reduction in tidal range due to the restriction (Table 1) and by the increase in marsh surface elevation due to introduced *P. australis* [95]. At one of our study sites (Hatches Harbor), Smith et al. [35] measured tidal range pre-restoration and found that tidal range in the restricted marsh was only 39% of that measured in the adjacent unrestricted marsh. At another one of our sites (Galilee), the depth and duration of flooding in the restricted marsh increased post-restoration, whereas the reference marsh remained the same [25]. Osgood et al. [96] found that a *P. australis*-invaded (unrestricted) marsh in Connecticut was 29.0 cm higher in elevation than an adjacent *S. alterniflora* marsh, resulting in a reduction in flooding frequency by 52%. Similarly, Hunter et al. [97] documented declines in marsh surface flooding depth from the initial (6.0±0.5 cm), early (3.9±1.3 cm), to late (2.4±0.8 cm) invasion stages that corresponded with reductions in flooding frequency by 7%, 16%, and 37%, respectively, in three *P. australis*-invaded (unrestricted) salt marshes in the mid-Atlantic region.

Our data suggest that with reduced or limited access to the marsh surface, *F. heteroclitus* in tidally restricted marshes invaded by *P. australis* are not obtaining dietary prey items needed to supplement their energy intake. Invertebrate prey on the marsh surface can differ than those typically found in subtidal creeks, with the former composed of isopods, gastropods, insects, spiders, beetles, amphipods, and ostracods and the latter composed of copepods, amphipods, and polychaetes [98]. The guts of fish allowed access to marsh surface can be up to six times fuller than those restricted to unvegetated subtidal creeks [98], providing resources necessary for significantly higher growth rates and weight gain [4], [12], [14], [15]. In unrestricted *P. australis* marshes in the Hudson River estuary, Weinstein et al. [16], [99] reported reductions in the energy reserves (triacylglycerols, free fatty acids, total lipids) of fish, which they attributed to reduced flooding frequency and access to the marsh surface for feeding. Therefore, decreased lipid reserves detected in our study could be due to lack of fish access to invertebrate prey on the marsh surface.

A second potential reason for reduced lipid reserves relates to increased movement of fish due to predation risk and reduced habitat refugia at high tide. For *F. heteroclitus*, predation risk is of primary importance in determining habitat use [45], [46], [100]. At low tide, *F. heteroclitus* will occupy depositional areas of water channels where prey is less abundant but predation pressure is low [45]. When the tide rises, fish flood onto the marsh surface to feed and escape predators [45], with adult *F. heteroclitus* moving farther onto the marsh surface than juveniles, which stay near the marsh fringe [19]. Increased risk of predation could confine movement of *F. heteroclitus* to areas with poor prey availability [45], decrease growth rates [46], or increase movements to avoid capture from predatory fish and wading birds [101], [102], thereby decreasing resources available for energy storage.

We found gravidity in *F. heteroclitus* strongly influenced their lipid reserves. Not only did we detect a significant cost of reproduction in *F. heteroclitus* (as evidenced by reduced lipid stores in unparasitized gravid females), the decreased proportion of gravid fish in restricted marshes suggests that investment tradeoffs between growth, lipid storage, and reproduction are occurring in the restricted marsh fish. Competing demands for energy acquisition, avoidance of predators while foraging, parasitism, and coping with seasonal fluctuations in north temperate estuaries influence energy allocation strategies in fish [49]–[51], [61]. Notably, we did not find any differences in growth rate or morphology between the restricted, restored, or reference marsh fish, indicating investment into growth is a high priority across all populations. Reproduction is costly [50], [92], so fish may choose to skip spawning and invest resources into growth and survival to enhance the chance of future success rather than deplete current lipid stores by spawning [50]. Whether decreased lipid reserves in unparasitized fish inhabiting restricted marshes were due to decreased foraging ability, increased movement due to predation, or some other factor, it appears that investment into lipid stores has been forgone in lieu of growth.

### Effectiveness of Tidal Restoration

Restoring hydrologic flow to salt marshes to decrease the cover and height of introduced *P. australis* has been a standard restoration practice in New England for decades and is used to re-establish habitat quality for salt marsh nekton and birds [24], [30]. Previous authors in New England have examined hydrologic restoration effectiveness using gut content analyses, nekton density, length frequency distributions, fish biomass, and species richness/diversity, with varying outcomes based on restoration longevity, tidal range, site location, species, and metric assessed [25], [34], [36], [37], [40], [43], [44], [67], [71], [77], [103]. Our results support the effectiveness of tidal restoration for nekton, as all environmental, physiological, and morphological indices revealed that hydrologically restored marshes were equivalent in habitat quality for fish relative to adjacent reference systems.

Notably, fish using the reference *S. alterniflora* marshes were smaller in length than those within the restored marshes, likely because we captured a significantly larger abundance of younger individuals (ages 0–1) in the reference marshes. Intertidal salt marshes serve as nurseries for young *F. heteroclitus* [3], [4], [19], [100], which use small surface marsh pools and depressions for feeding and refuge during their first summer until they have obtained sufficient length to enter the tidal creek system [32], [33], [96], [97]. Many of our restored sites are still changing and have yet to develop an extensive network of pools typical of salt marshes, so exposure of juveniles to predators may be higher than in reference marshes. Adult *F. heteroclitus* are known to consume their younger conspecifics so it possible that young-of-the-year fish are fewer in number in restored marshes simply due to predation [33], [102].

Over time, nekton patterns in the restored marshes can mimic those in reference areas as the hydrologic connection between habitats allows greater faunal and prey exchange [34], [37], [40], [44]. Our results demonstrate that restored and reference salt marshes are equivalent in their provision of habitat to resident salt marsh fish as indicated by non-significant differences in energy reserves, growth rate, morphology, gravidity, parasite prevalence, and water quality 11–22 years post-restoration. The outcomes of our study agree with the findings of two recent meta-analyses (one global, one regional) that concluded that in degraded wetlands ecological restoration of faunal communities can rapidly occur within the timeframe of 5–10 years, especially where there is a hydrologic connection to an intact marsh system [104, K.L. Dibble, unpublished data]. Although other wetland functions such carbon storage and nutrient cycling may take many more years to achieve [104], habitat quality for fauna can be restored relatively quickly in these systems.

### Conclusions

Tidally restricted salt marshes invaded by introduced *P. australis* have been the focus of restoration efforts due to measurable differences in biodiversity and ecosystem function. We demonstrated that fish in restricted, restored and reference marshes are morphologically similar so that an assessment of condition based on fish length or biomass might not capture the physiological effects of poor habitat quality on resident fish populations. Instead, we used biochemical condition indices and examined parasites and gravidity and were able to detect trends in the health of a common marsh resident. Numerically dominant along the Atlantic coast, *F. heteroclitus* consume salt marsh herbivores/detritivores and are prey to transient predators, thereby providing an important trophic link between intertidal marsh production and near- and offshore food webs [102]. Management efforts to restore tidal exchange and control the *P. australis* invasion in salt marshes should be a priority to ensure that forage fish populations are healthy and can support coastal fisheries.
